# Noninvasive prenatal testing in the general obstetric population: clinical performance and counseling considerations in over 85 000 cases†

**DOI:** 10.1002/pd.4766

**Published:** 2016-01-27

**Authors:** Patricia A. Taneja, Holly L. Snyder, Eileen de Feo, Kristina M. Kruglyak, Meredith Halks‐Miller, Kirsten J. Curnow, Sucheta Bhatt

**Affiliations:** ^1^ IlluminaRedwood CityCAUSA

## Abstract

**Objective:**

The primary goal of this study was to provide clinically relevant information for appropriate patient counseling.

**Method:**

Demographics and test metrics were reviewed for 86 658 clinical cases. Outcome information was requested for samples reported as aneuploidy detected or suspected for chromosomes 21, 18, or 13; voluntary outcome reporting was encouraged for all discordant outcomes.

**Results:**

Of 86 658 cases, 85 298 (98.4%) met inclusion criteria for result reporting. Of the 1360 (1.6%) cancellations, only 101 (0.1%) were for technical reasons. Average time to result was 3.3 business days. Aneuploidy was detected or suspected in 2142 (2.5%) samples. For aneuploidy detected cases with known clinical outcomes, the overall positive predictive value (PPV) was 83.5% (608/728); observed PPVs for trisomies 21, 18, and 13 ranged from 50.0 to 92.8%. As individual PPVs are determined by a patient's prior risk, we developed a chart for counseling patients on positive predictive value based on maternal age.

**Conclusion:**

This large‐scale report reinforces that noninvasive prenatal testing is a highly accurate screen for fetal aneuploidy in the general obstetric population. Test improvements have facilitated a reduction in failure rates, time to result, and borderline results/unclassifiable results. We have developed a positive predictive value counseling tool to ensure appropriate patient education, counseling, and clinical utilization. © 2015 Illumina. *Prenatal Diagnosis* published by John Wiley & Sons, Ltd.

## Abstract 2

**What's already known about this topic?**

- Noninvasive prenatal testing (NIPT) has been shown to screen for common fetal aneuploidies with high sensitivity and low false positive rates.
- NIPT is a reliable alternative to current fetal aneuploidy serum screening methods in the first and second trimesters.
- Previous publications detailing NIPT clinical experience have shown that NIPT is performing as well as it performed in clinical validation studies.

**What does this study add?**

- Analysis of over 85 000 samples submitted to the clinical laboratory suggests that whole genome sequencing‐based NIPT continues to meet or exceed performance characteristics established by clinical validation studies for screening of fetal aneuploidy.
- A tool to guide appropriate pre‐test and post-test counseling of patients on estimated positive predictive values based on their personal maternal-age based risk, with recommendations for effective implementation into clinical practice.

## Introduction

There has been exponential growth in the uptake of noninvasive prenatal testing (NIPT) since initial clinical validation studies demonstrated that whole genome massively parallel sequencing of cell‐free DNA (cfDNA) can detect fetal aneuploidy with high accuracy.^1^, ^2^, ^3^, ^4^ In response to the introduction of cfDNA technology, several medical societies published policy statements about the use of NIPT.^5^, ^6^, ^7^, ^8^, ^9^ Statements from the International Society of Prenatal Diagnosis^9^ and the American College of Medical Genetics and Genomics^6^ include a recommendation for ongoing reporting of clinically relevant metrics, such as test performance, failure rates, and turnaround time. Futch *et al*. published the initial clinical experience of the Illumina clinical laboratory from nearly 6000 high‐risk pregnancies.^10^ Our clinical experience with sex chromosome analysis was also detailed in a recent publication.^11^ Both studies indicated that clinical cfDNA testing operated well within the performance parameters established in prior validation studies. Additionally, other clinical laboratories^12^, ^13^ and individual clinics^14^, ^15^, ^16^, ^17^ have published their clinical experience with cfDNA testing. These publications help to monitor individual clinical laboratory metrics and point out emerging trends and challenges within the broader field of NIPT. The focus of recent NIPT‐related publications has shifted towards providing clinicians with pre‐test and post‐test counseling tools.^18^, ^19^

This study had two main goals. First, determine updated performance metrics and then develop a positive predictive value (PPV) counseling tool that links NIPT clinical performance to an *a priori* risk determined by maternal age. Second, evaluate whether the clinical population demographics changed after test introduction.^10^

## Methods

This study was a retrospective analysis of data prospectively collected or generated on consecutive clinical samples submitted for the verifi prenatal aneuploidy screening test at the College of American Pathologists‐accredited and Clinical Laboratory Improvement Act‐certified Illumina Laboratory (Verinata Health, Inc., a wholly owned subsidiary of Illumina, Inc., Redwood City, CA). This test screens for fetal autosome aneuploidy (chromosomes 13, 18, and 21) by analyzing cfDNA via massively parallel sequencing. Within the timeframe of this study, several process improvements and analytic updates to the test were implemented after internal analytical validation. This study cohort included all singleton pregnancy clinical samples tested for autosomal trisomies on chromosomes 21, 13, and 18, subsequent to those previously published.^10^ Samples reported as a single autosome monosomy or multiple detected aneuploidies were also excluded from this study. For a subcohort of cases described here, results from sex chromosome analysis were published separately.^11^

Noninvasive prenatal screening was performed as previously described.^11^ Samples could be canceled because of either administrative^10^ or technical reasons. Technical cancellations were samples that did not meet quality control (QC) standards and included high cfDNA (i.e., cfDNA extraction quantification returns a value greater than our acceptable internal QC limit), insufficient cfDNA content (i.e., library preparation quantification returns a value less than our acceptable internal QC limit), QC failure (i.e., final analysis metrics do not meet the acceptable values set forth in our internal QC standards), and laboratory processing issue (i.e., samples cannot continue in the process because of an issue that is not related to QC failures such as sample drop or centrifugation error). Administrative cancelations did not begin the testing process. Common reasons for administrative cancelations included insufficient sample quantity, tube received beyond stability period (>5 days from draw), test canceled by ordering physician, and gestational age of less than 10 weeks.

Providers were notified if the test was canceled and offered the option to submit a second sample. Samples completing the test process were categorized as no aneuploidy detected (NAD), aneuploidy detected (AD), or aneuploidy suspected (AS). The AS cases fall in the borderline zone between the overlapping bimodal distributions of AD and NAD populations.

An active follow‐up process (fax and phone)^10^ was utilized to collect outcome information for cases with AD and AS results for trisomy 21 (T21), trisomy 18 (T18), or trisomy 13 (T13) as well as technical cancelations, according to standard laboratory practice and quality procedures as previously described.^11^ Cases were categorized as follows: (1) ‘concordant with karyotype’ if NIPT results matched a karyotype or physical exam (true positive, TP); (2) ‘concordant with no karyotype’ if no karyotype was known to the laboratory, but ultrasound findings or other risk indications were suggestive of aneuploidy (soft markers on ultrasound and positive serum screening results were not considered suggestive of aneuploidy); (3) ‘pregnancy loss’ if a spontaneous miscarriage or fetal demise occurred without confirmatory karyotype analysis; (4)‘discordant’ if NIPT results did not match karyotype or birth outcome (false positive, FP) or for NAD cases, where follow‐up was not actively carried out but outcomes were accepted if reported (false negative, FN); or (5) ‘no information’ if outcome information was unavailable.

For clinical outcome data, observed PPV was calculated from cases with known cytogenetic outcomes [(TP)/(TP + FP)]. The PPV counseling chart, which shows projected PPVs for each indication by maternal age, was calculated from assay sensitivities and specificities and published estimates of incidence at 10 weeks of gestation^20^ using the following equation: (Incidence × Sensitivity)/{[Incidence x Sensitivity]+[(1 − Incidence) × (1 − Specificity)]}. Performance metric calculations are described in more detail in Supplement 1.

Where possible, demographic and result data were compared between the current study population and our initial clinical experience.^10^ Statistical significance was determined by an unpaired *t*‐test for continuous variables and by a chi‐squared test for categorical variables. A *p*‐value less than 0.05 was considered significant. Analyses were performed using the R statistical package (version 2.12.0).

## Results

### Laboratory experience

**Figure 1:**
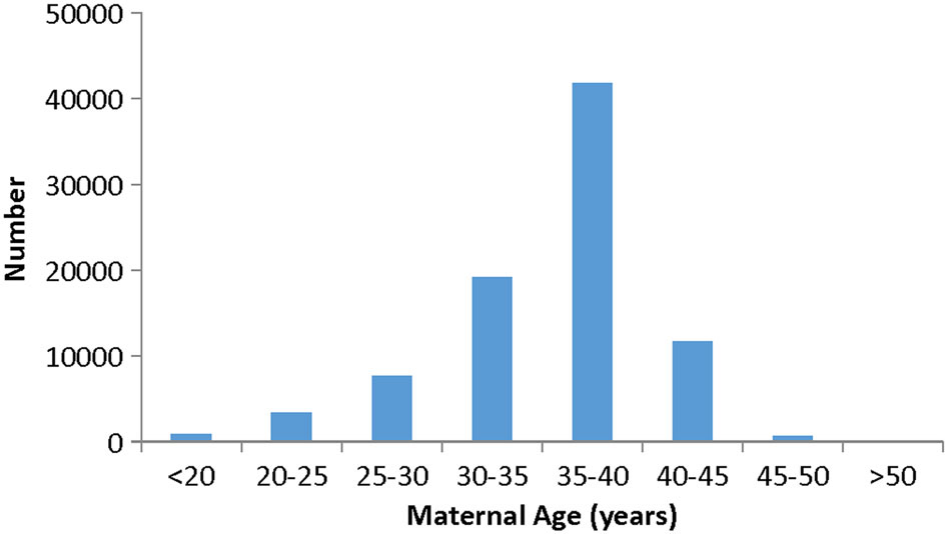
Maternal age histogram for clinical cohort

A total of 86 658 samples meeting inclusion criteria were accessioned during the study period. Samples were received from across the United States and 38 different countries. Test metrics and demographic characteristics are shown in Table 1 and compared with our initial clinical population.^10^ The maternal age distribution is shown in Figure 1; average maternal age was not significantly different between the two study cohorts (*p* = 0.053). There has been a shift in the test timing, with testing now predominantly performed in the first trimester (63.5% vs 47.2%; *p* < 0.0001) as compared with the second/third trimesters (36.5% vs 52.8%; *p* < 0.0001). Process improvements have led to a 30% reduction in time to result (turnaround time; *p* < 0.0001), now 3.3 business days from receipt of sample to reporting, and an 86% reduction in the technical test cancellation rate to 0.1% (*p* <0.0001; Table 1).

**Table 1:** Demographic and test metric comparison between clinical cohorts

Variable	CLIA laboratory *n* = 86 658^a^	Futch *et al*.^10^ *n* = 6123^b^
Maternal age (years)		
*n*	85 200	6123
Mean ± SD	35.3 + 5.1	35.0 ± 5.7
Min–max	13.8–57.8^c^	14.6–51.7
Gestational age (weeks)		
*n*	85 144	6123
Mean ± SD	14.0 ± 4.2	15.6 ± 4.6
Min–max	4^d^–38	5^d^–37
Gestational age group, *n* (%)^e^		
*n*	85 144	6123
First (10–13.9 weeks)	54 088 (63.5)	2883 (47.2)
Second (14–27.9 weeks)	29 963 (35.2)	3103 (50.8)
Third (28–40+ weeks)	1093 (1.3)	127 (2.1)
Turnaround time (business days)		
Mean	3.3	5.1
Interquartile range	2–4	4–6
Total cancelations, *n* (%)	1376 (1.6)	149 (2.4)
Technical^f^	101 (0.1)	43 (0.7)
Administrative^g^	1214 (1.4)	106 (1.7)
Site‐specific^h^	45 (0.05)	

CLIA, Clinical Laboratory Improvement Amendments.

a Not all samples reported all demographic variables; thus, counts are reported separately.

b Data from Futch T., Spinosa J., Bhatt S., De Feo E., Rava R.P., and Sehnert A.J. Initial clinical laboratory experience in noninvasive prenatal testing for fetal aneuploidy from maternal plasma DNA samples. Prenat Diagn 2013; 33:569‐74.

c Maternal age was confirmed in both low and high extremes; the maternal age range was similar to other large‐scale clinical studies.^12^, ^21^

d Samples received from patients under 10 weeks of gestational age were canceled.

e Trimester at time of blood draw.

f Technical cancelations are samples that did not meet quality control standards; this included high cfDNA (41/101, 58.9%), insufficient cfDNA content (11/101, 7.5%), quality control (QC) failure (43/101, 29.5%), and laboratory processing issue (6/101, 4.1%).

g Administrative cancelations did not begin the testing process. Common reasons for administrative cancelations were insufficient sample quantity, tube received beyond stability period (>5 days from draw), test canceled by ordering physician, and gestational age less than 10 weeks.

h Cancelations were because of an international site‐specific sample stability issue that has since been resolved.

### Aneuploidy reporting

Results of testing are shown in Table 2. Of 2142 (2.5%) positive results, the majority (86.7%, 1858/2142) were AD. The overall incidence of positive cases (AD/AS) has declined from 6.9% in the Futch *et al*. cohort^10^ to 2.5% (Table 2). This reflects a significant reduction in the prevalence of AD cases, 4.0% to 2.2%, and a significant reduction in the prevalence of AS cases, 2.8% to 0.3%.

**Table 2:** Comparison of aneuploidy incidence between study cohorts

Variable	CLIA laboratory	Futch *et al.* 10	*p*‐value
Reported cases, n	85 298	5974	
No aneuploidy detected, n (%)	83 156 (97.5)	5564 (93.1)	<0.0001
Aneuploidy detected (AD), *n* (%)	1858 (2.2)	240 (4.0)	<0.0001
Chromosome 21, *n* (%)	1255 (1.5)	155 (2.6)	
Chromosome 18, *n* (%)	412 (0.5)	66 (1.1)	
Chromosome 13, *n* (%)	191 (0.2)	19 (0.3)	
Aneuploidy suspected (AS), *n* (%)^a^	284 (0.3)	170 (2.8)	<0.0001
Chromosome 21, *n* (%)	102 (0.1)	60 (1.0)	
Chromosome 18, *n* (%)	136 (0.2)	50 (0.8)	
Chromosome 13, *n* (%)	46 (0.05)	60 (1.0)	

CLIA, Clinical Laboratory Improvement Amendments.

a AS cases were denoted as ‘unclassifiable’ in the original publication (Futch *et al*.)^10^

### Outcome information

Outcomes were requested for all AD/AS cases. Of the 1197 responses received (55.9%), 1094 (91.4%) provided informative outcome information (e.g., karyotype, abnormal ultrasound findings, or pregnancy loss) and 103 (8.6%) responded but had no informative outcome information. A number of laboratories and providers opted out of providing follow‐up which led to 356 samples where information was not requested. Additionally, information was requested but was not received for a further 589 cases.

**Figure 2:**
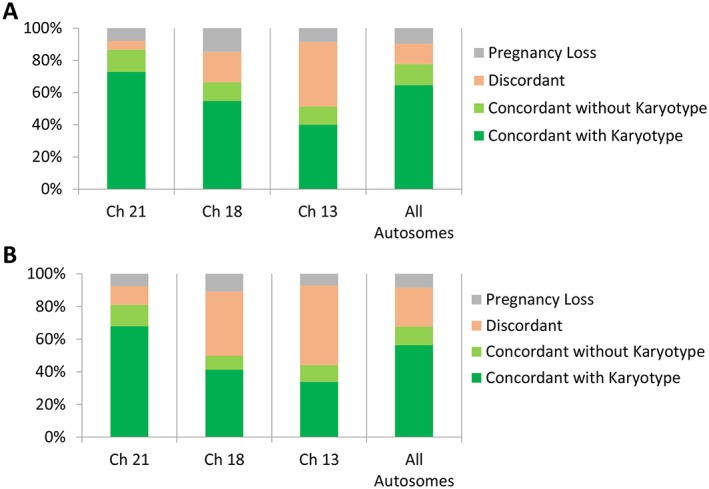
Informative clinical outcomes. (A) Aneuploidy detected cases (*n* = 940/1858). (B) All positive results (aneuploidy detected and aneuploidy suspected, *n* = 1094/2142). Ch, chromosome

Within the 1858 AD cases, 940 (50.6%) had outcomes (Figure 2A); 608 (439 T21, 127 T18, and 42 T13) were confirmed by cytogenetic studies, 122 (83 T21, 27 T18, and 12 T13) were concordant based on clinical findings highly suspicious of aneuploidy (e.g., abnormal ultrasound findings) but lacked karyotype information, 120 (34 T21, 44 T18, and 42 T13) had discordant (FP) clinical outcomes, and 90 had a pregnancy loss without karyotype analysis. For the 2142 AD/AS cases as a whole, 1094 (51.1%) had outcomes (Figure 2B); 616 AD/AS results were confirmed by cytogenetic studies, 125 were concordant with no karyotype information, 261 cases had discordant outcomes, and 92 had a pregnancy loss without karyotype analysis.

Within the 85 298 reported cases, the observed false–positive frequency was 0.1% (120/85 298) for AD cases, and 0.3% (261/85 298) for the AD/AS cases as a whole. Overall, as expected, there was a higher concordance within the AD cases, which comprise the majority of the positive samples. Of the 85 298 reported cases, the laboratory was notified of 15 (0.02%) FNs, including six cases of T21 (including one fetal mosaic), seven cases of T18, one case of T13, and one case of fetal mosaicism for both T13 and T18; maternal age and gestational age characteristics for FNs (Table S1) were similar to the overall cohort (Table 1).

### Clinical performance metrics and positive predictive value counseling tool

Observed performance statistics were derived based on available outcome data (Table 3), with the cohort size adjusted for the proportion of positive cases with confirmed outcomes (cohort adjustment calculations are detailed in Supplement 1). Because complete outcomes were not available, sensitivity and specificity ranges were estimated by assuming that positive samples lacking outcomes were all concordant (upper limit) or all discordant (lower limit). For these calculations, samples that were reported as ‘NAD’ by NIPT and that had no further communication regarding discordant outcomes were considered to be true negatives.

**Table 3:** Sensitivity and specificity for AD/AS samples compared with published validation metrics

Indication	CLIA laboratory				Validation studies a	
	Observed sensitivity b	Sensitivity range c	Observed specificity^b^	Specificity range^c^	Sensitivity	Specificity
Trisomy 21	99.49%	98.66–99.53%	99.77%	98.92–99.91%	100%	99.76%
Trisomy 18	97.23%	94.20–98.15%	99.69%	99.51–99.85%	97.37%	99.57%
Trisomy 13	97.98%	95.56–98.87%	99.84%	99.77–99.93%	87.50%	100%

a Validation performance from the MELISSA cohort,^4^ which included 90 trisomy 21 samples, 38 trisomy 18 samples, and 16 trisomy 13 samples; unclassified samples were treated as positives (Supplement 1).

b Observed sensitivity and specificities were calculated using available outcome data with the cohort size adjusted for the proportion of positive cases with confirmed outcomes.

c The low end of the range was based on the assumption that all unreported outcomes are discordant, and high end of the range was based on the assumption that all unreported outcomes are concordant.

**Figure 3:**
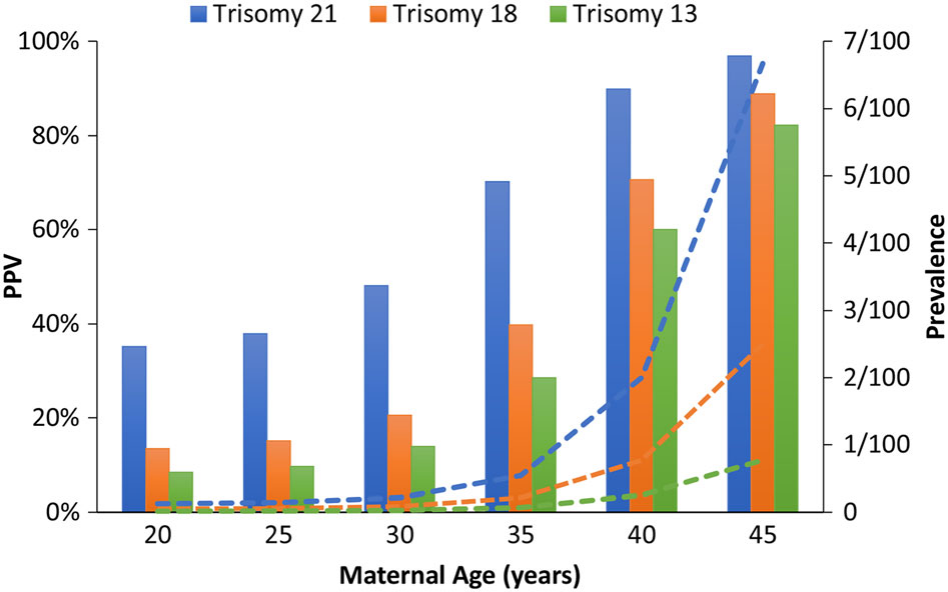
Positive predictive value counseling tool. Positive predictive values (bars) based on estimated prevalences at 10 weeks of gestation^20^ (dashed lines) by maternal age and observed sensitivities and specificities (Table 3). PPV, positive predictive value

Observed PPVs were derived based on cases with cytogenetic confirmation (Table 4). In this study, the observed per chromosome PPVs for AD cases ranged from 50.0% to 92.8%. While overall PPVs were high, an individual patient's PPV is dependent on their personal *a priori* risk, which reflects a combination of maternal age, gestational age, and the presence or absence of other indications of fetal aneuploidy. For women undergoing NIPT as a first‐tier screen, maternal age is the primary factor determining *a priori* risk. By combining the observed sensitivities and specificities (Table 3) determined here with published estimates of incidence at 10 weeks of gestation (Table S2),^20^ we projected PPVs for T21, T18, and T13 at five‐year maternal age intervals (Figure 3), demonstrating that later maternal ages have higher PPVs because of the higher incidence of fetal aneuploidy.

**Table 4:** Observed positive predictive values by condition

Variable	Trisomy 21	Trisomy 18	Trisomy 13	Overall
AD/AS samples a	85.5% (443/518)	51.2% (130/254)	41.0% (43/105)	70.2% (616/877)
AD samples a	92.8% (439/473)	74.3% (127/171)	50.0% (42/84)	83.5% (608/728)

a Observed PPV based on cytogenetically confirmed cases.

## Discussion

The increasing clinical utilization of NIPT within the general prenatal screening population has prompted considerable discussion focused on the importance of communicating appropriate test metrics. In addition, several professional societies have called for ongoing reporting of NIPT clinical test performance metrics.^5^, ^6^, ^7^, ^9^, ^22^ As this is still a relatively new area in the prenatal field and is an area where available assays are continually updated and improved upon, we believe that it is important to continue to report on the current performance metrics of these NIPT assays. Additionally, as NIPT moves from being a high‐risk pregnancy screening test to a screening tool for both high‐risk and low‐risk women, it is important that the performance of the test in this changing patient population is reported. Since the first clinical experience publication,^10^ this NIPT has undergone several important validated updates of sequence chemistry and proprietary algorithms, which were intended to reduce failure rates and false–positive rates of the test; these updates have resulted in improved performance of the test offered in the clinical laboratory. Here, we compared our recent clinical experience with our initial experience to communicate these improvements in test performance and describe the change in the clinical population of patients undergoing NIPT since its introduction. Further, we used these updated metrics to develop a PPV clinical counseling tool.

This study demonstrated improvements in three key performance metrics: Time to result, cancelation rates, and borderline result classification. Here, we show that the technical cancelation rate has been reduced from 0.7% to 0.1% with the implementation of test improvements. Importantly, this technical cancelation rate (0.1%) is far below those reported by other NIPT laboratories (1.9–7.7%).^1^, ^12^, ^13^ Further, clinical follow‐up of this small technical cancelation group revealed no aneuploidies (0/52) for chromosomes 13, 18, or 21 within the cases that reported back outcomes to the laboratory (52/101, 51.5%). This contrasts with two recent studies from other laboratories that suggested aneuploidy cases are over‐represented in technical cancellations.^23^, ^24^ These conflicting findings may reflect differences in assay design, chemistry, and/or bioinformatics analysis methods between whole genome sequencing approaches and targeted sequencing approaches. In a clinical setting, canceled tests generally lead to inconvenient second blood draw appointments, increased turnaround times, and heightened patient anxiety. Thus, with this NIPT, the vast majority of patients receive results, and based on the above data, a cancelation does not elevate a patient's *a priori* risk for fetal aneuploidy.

Since its introduction in 2012, NIPT has been rapidly adopted into clinical practice. The initial NIPT clinical offering focused almost exclusively on high‐risk women and often as a second‐tier screening option, resulting in a high AD incidence and a significant proportion of second trimester samples.^10^ We evaluated whether the clinical population opting for NIPT has changed since its introduction. This study showed that there has been a shift towards first trimester use, consistent with greater utilization of NIPT as an earlier first‐tier aneuploidy screen. There has also been a significant decrease in the overall prevalence of positive (AD/AS) cases reported by the laboratory.^10^ This is attributed to a combination of two factors. First, the lower overall prevalence suggests changing indications, with more patients without clearly defined high‐risk indications choosing NIPT. Indications from the test requisition forms suggest that the current study had a higher proportion of low‐risk patients (data not shown), including patients with milder or no ultrasound findings compared with the study by Futch *et al*. Unfortunately, as indications on the test requisition form are not completed by all providers, we are unable to definitely say what the different risks in the two populations are. No shift in prevalence was noted for T13, which could be because of the relatively low overall incidence of T13, even in a high‐risk population. Second, advances in sequencing chemistry and the analysis algorithm have facilitated a greater refinement of the borderline zone between NAD and AD, reducing AS results. This improvement is of significant clinical value.

Increasing utilization of NIPT has highlighted the importance of evaluating and communicating clinical performance and test limitations. In this study, outcomes were not available for all cases, but observed sensitivities and specificities were in line with validation studies (Table 3), supporting that NIPT has maintained high levels of accuracy in a clinical setting. However, while NIPT has high sensitivities and specificities, it is important to recognize that FPs and FNs can occur. As such, all positive results should be confirmed by diagnostic testing. For AD cases in this cohort, the overall observed frequency of putative FPs was 0.1%, a small reduction compared with our initial clinical experience (0.2%).^10^ This study cohort had a reported overall false–negative frequency of 0.02%, which is comparable to other published reported false‐negative frequencies [0.01–0.06%].^12^, ^13^, ^17^ As false–negative results are based on cases that were self‐reported to the laboratory only, the true false–negative value may be higher.

One of the biggest challenges surrounding NIPT has been understanding test performance statistics and how to apply them to specific patient populations, particularly with the increasing adoption of NIPT in women with a lower *a priori* risk. As a result, there has been a shift in recent studies to reporting predictive values^12^, ^13^, ^24^, ^25^ because predictive values can be more useful when counseling patients. In this study, the observed per chromosome PPVs for AD cases ranged from 50.0% to 92.8% (Table 4), consistent with other published NIPT PPVs.^12^, ^13^, ^24^ The lower PPVs for chromosome 13 and 18 were expected, as T18 and T13 have a lower incidence than T21 and more cases of fetal and placental mosaicism have been reported for chromosomes 13 and 18.^26^

While there has been a push from professional societies to move to reporting PPVs on NIPT reports delivered to patients,^19^, ^27^ this has not yet been adopted. The primary reason is likely in part because of the dependency of PPVs on an *a priori* risk, which makes reporting a personalized PPV difficult. A patient's *a priori* risk depends on a combination of variables, including maternal age, gestational age, family history, and the presence of other high‐risk indications (e.g., ultrasound findings or positive serum screening results). Unfortunately, detailed patient information is not always provided on the test requisition form which can increase the difficulty of personalized PPV reporting. To aid counseling for patients with a positive result, we developed a PPV chart (Figure 3) that can be used by clinicians as a guide to a patient's PPV based on maternal age alone. When counseling patients, clinical consideration should be given to the presence of other indications (e.g., ultrasound findings) that may elevate a patient's *a priori* risk, and therefore PPV, over that determined by maternal age alone. Women considered to be low risk (no known high‐risk indications) should be counseled that they will have a lower PPV. Although the PPV for low‐risk women is lower than for high‐risk women, it is important for clinicians to understand that the PPV for NIPT is higher than with traditional pregnancy screening options, regardless of maternal age or *a priori* risk.^24^, ^25^ We recommend that this PPV tool is used in clinical practice to better inform patients of their risk; however, diagnostic invasive testing is always recommended for confirmation of a high‐risk NIPT result. It is also important for clinicians to note that, as PPVs vary based on the NIPT assay, the PPV tool outlined in this study is specific to the verifi NIPT only.

One of the limitations of this study was incomplete clinical outcomes. Obtaining clinical outcomes remains a challenge for all NIPT laboratories.^10^, ^12^, ^14^, ^15^ There are several factors that may contribute to incomplete outcomes, including the absence of a clinical point person at the draw location (healthcare provider or distributor laboratory) to communicate this information back to the laboratory, patients that move or transfer care, a dependence on providers to report putative false‐negative results, and ethical concerns of providers regarding the discussion of patient information. Even with an active outcome request protocol for AD and AS reports and technical cancelations, there was still some difficulty obtaining this information from clinicians. Thus, the potential ranges were determined for sensitivity and specificity. This is in contrast to other clinical outcome studies with incomplete outcomes, where sensitivities and specificities were either not reported^12^ or were reported as point estimates that are likely to be inflated because unknown outcomes were assumed to be concordant (equivalent to the upper limit detailed here).^13^ It is anticipated that the true test performance is somewhere between the observed level and upper limit, because many unconfirmed outcomes were cases that lacked karyotype confirmation but had clinical findings suggestive of aneuploidy.

## Conclusion

As more general obstetric population studies are published and as NIPT expands to include additional chromosome and microdeletion analysis, continued updates on clinical laboratory experience will remain necessary to ensure that patients have appropriate resources when facing decisions regarding diagnostic invasive prenatal tests. This includes appropriate counseling regarding test performance statistics and population statistics. When interpreting PPVs, the commonly reported clinical performance metric, it is important for clinicians to understand that PPVs change with aneuploidy incidence, so as the population incidence decreases, PPVs will as well. Patients receiving an aneuploidy detected or suspected result via NIPT should receive post‐test counseling to assess their individual clinical picture and be offered standard confirmatory diagnostic testing.^5^, ^6^, ^7^, ^19^, ^22^, ^27^, ^28^, ^29^ Irreversible clinical decisions should not be made based on screening results alone.^27^ For patients with discordance, clinicians should consider potential biological etiologies (e.g., CPM, fetal mosaicism, and maternal medical conditions), and depending on the individual clinical picture, consider whether further clinical follow‐up is warranted.

**What's Already Known About This Topic?**

- Noninvasive prenatal testing (NIPT) has been shown to screen for common fetal aneuploidies with high sensitivity and low false positive rates.
- NIPT is a reliable alternative to current fetal aneuploidy serum screening methods in the first and second trimesters.
- Previous publications detailing NIPT clinical experience have shown that NIPT is performing as well as it performed in clinical validation studies.

**What Does This Study Add?**

- Analysis of over 85 000 samples submitted to the clinical laboratory suggests that whole genome sequencing-based NIPT continues to meet or exceed performance characteristics established by clinical validation studies for screening of fetal aneuploidy.
- A tool to guide appropriate pre-test and post-test counseling of patients on estimated positive predictive values based on their personal maternal-age based risk, with recommendations for effective implementation into clinical practice.

## Supporting information

Supporting info item
