# Supplementary material for: Noninvasive prenatal testing in the general obstetric population: clinical performance and counseling considerations in over 85 000 cases
Source: Prenat Diagn. 2016 Jan 27;36(3):237–43. doi: 10.1002/pd.4766 (PMC4819889; doi:10.1002/pd.4766)
Supplement: Supplementary file 1 — Supporting info item [file PD-36-237-s001.docx]

**Supplement 1. Performance Metrics Calculations**

The sensitivities and specificities reported in the MELISSA study^4^ were for aneuploidy detected samples (unclassified samples excluded), and were as follows: Trisomy 21, 100% (89/89) sensitivity and 100% (404/404) specificity; Trisomy 18, 97.2% (35/36) sensitivity and 100% (460/460) specificity; Trisomy 13, 78.6% (11/14) sensitivity and 100% (485/485) specificity. However, in a clinical setting, unclassified samples have been reclassified as aneuploidy suspected. The performance metrics when both aneuploidy detected and aneuploidy suspected results are considered as positive results are: Trisomy 21, 100% (90/90) sensitivity and 99.8% (409/410) specificity; Trisomy 18, 97.4% (37/38) sensitivity and 99.6% (461/463) specificity; Trisomy 13, 87.50% (14/16) sensitivity and 100% (485/485) specificity; these are the performance metrics included on verifi test requisition forms and patient reports, and reflect results from analysis of the MELISSA cohort using updated sequencing chemistry and an optimized algorithm.

Observed sensitivities and specificities were derived based on available outcome data, which required adjustment of the cohort size to account for the incomplete outcomes. For trisomy 21, 1,356 cases were reported as positive and 83,942 were reported as negative: cytogenetic confirmation revealed 443 TP, 75 FP, and 6 FN; the proportion of positive cases with outcomes was 38.2% = (443 + 75) / 1,356, so the adjusted FN number was 2 = 6 x 38.2%, and the adjusted TN was 32,064 = (83,942 - 6) x 38.2%; observed sensitivity was 99.6% = 443 / (443 + 2) and observed specificity was 99.8% = 32,064 / (32,064 + 75). For trisomy 18, 549 cases were reported as positive and 84,749 were reported as negative: cytogenetic confirmation revealed 130 TP, 124 FP, and 8 FN; the proportion of positive cases with outcomes was 46.3% = (130 + 124) / 549, so the adjusted FN number was 4 = 8 x 46.3%, and the adjusted TN was 39,206 = (84,749 - 8) x 46.3%; observed sensitivity was 97.0% = 130 / (130 + 4) and observed specificity was 99.7% = 39,206 / (39,206 + 124). For trisomy 13, 237 cases were reported as positive and 85,061 were reported as negative: cytogenetic confirmation revealed 43 TP, 62 FP, and 2 FN; the proportion of positive cases with outcomes was 44.3% = (43 + 62) / 237, so the adjusted FN number was 1 = 2 x 44.3%, and the adjusted TN was 37,684 = (85,061 - 2) x 44.3%; observed sensitivity was 97.7% = 43 / (43 + 1) and observed specificity was 99.8% = 37,684 / (37,684 + 62). Any differences between the observed sensitivities and specificities detailed above and Table 3 are due to rounding of the adjusted FN and adjusted TN in the text explanation.

The PPV counseling chart (Figure 2) was calculated from observed sensitivities and observed specificities determined here and published estimates of prevalence at 10 weeks of gestation[^21^](#_ENREF_21) (Table S2) using the following equation: (Incidence x Observed Sensitivity) / ([Incidence x Observed Sensitivity] + [{1 - Incidence} x {1 - Observed Specificity}]). For example, the PPV for a positive trisomy 21 result in a 20-year old patient is 35.1% = (1/800 x 99.49%) / ([1/800 x 99.49%] + [{1-1/800} x {100% - 99.77%}]).

**Table S1.** Demographic information for false negative cases

| **Outcome** | **Maternal Age (years)** | **Gestational Age (weeks)** |
| --- | --- | --- |
| 47,XX,+13[17]/47,XX,+18[3] | 42.8 | 10 |
| 47,XY,+13 | 36.0 | 12 |
| 47,XY,+18 | 42.8 | 10 |
| 47,X?,+18^a^ | 39.5 | 17 |
| 47,XX,+18 | 38.3 | 19 |
| 47,XX,+18 | 24.1 | 23 |
| 47,XY,+18 | 37.3 | 13 |
| 47,XX,+18 | 39.7 | 10 |
| 47,XX,+18 | 35.7 | 10 |
| 47,XX,+21 | 33.0 | 22 |
| 47,XX,+21 (mosaic)^b^ | 24.1 | 20 |
| 47,XY,+21 | 36.7 | 12 |
| 47,XY,+21 | 37.6 | 11 |
| 47,XY,+21 | 42.0 | 12 |
| 47,XY,+21 | 36.6 | 12 |

^a^ Gender not reported to laboratory

^b^ Outcome reported as mosaic, no cell count provided

**Table S2.** Estimated incidence of trisomy 21, trisomy 18, and trisomy 13 at 10 weeks of gestation.

|  | **Maternal Age (years)** | | | | | |
| --- | --- | --- | --- | --- | --- | --- |
| **Indication** | **20 years** | **25 years** | **30 years** | **35 years** | **40 years** | **44 years** |
| Trisomy 21 | 1/800 | 1/710 | 1/470 | 1/185 | 1/50 | 1/15 |
| Trisomy 18 | 1/2000 | 1/1750 | 1/1200 | 1/470 | 1/130 | 1/40 |
| Trisomy 13 | 1/6500 | 1/5600 | 1/3700 | 1/1500 | 1/400 | 1/130 |

Data from Gardner RJM, Sutherland GR, Shaffer LG. Parental age counseling and screening for fetal trisomy. *Chromosome abnormalities and genetic counseling*: Oxford University Press, 2012.
